# Multiple Tarsometatarsal Coalitions: A Case Report

**DOI:** 10.7759/cureus.22358

**Published:** 2022-02-18

**Authors:** Arshad Bashir, Muhammad A Hamid, Mudasir A Parry

**Affiliations:** 1 Department of Orthopaedic Surgery, Government Medical College, Srinagar, Srinagar, IND

**Keywords:** foot pain, midfoot sprain, foot and ankle surgery, tarsal coalition, tarsometatarsal coalition

## Abstract

Tarsometatarsal coalitions have rarely been reported in published literature. The few reported cases presented with varying degrees of pain. Here, we describe the case of a 16-year-old female with multiple tarsometatarsal coalitions, the first of its kind in the reported literature. Descriptions in anthropology literature suggest that these lesions might be more common than previously thought and, in some circumstances, can become symptomatic enough to warrant intervention.

## Introduction

Tarsometatarsal coalitions are rare in clinical practice, and not many cases have been reported in the published literature [1-5]. The ones published have reported only isolated coalitions of a metatarsal with one of the cuneiform bones or with the cuboid [6]. Here, we report the case of a 16-year-old female with symptomatic fifth metatarsal-cuboid and multiple cuneometatarsal coalitions of her right foot.

To our knowledge, our report is the only such case published in the orthopedic literature. We discuss the clinical relevance of this finding and the similarities with existing orthopedic and anthropology literature.

## Case presentation

A 16-year-old female presented to our outpatient clinic with complaints of pain on the outer aspect of her right foot for the past few weeks. She had suffered a twisting injury while walking downstairs and had been symptomatic since.

Our examination revealed a normal foot, comparable to the left side, and no signs of instability of either the ankle or foot. There were no signs of peroneal spasticity, and her arches were normal. There was no significant orthopedic or medical history.

**Figure 1:**
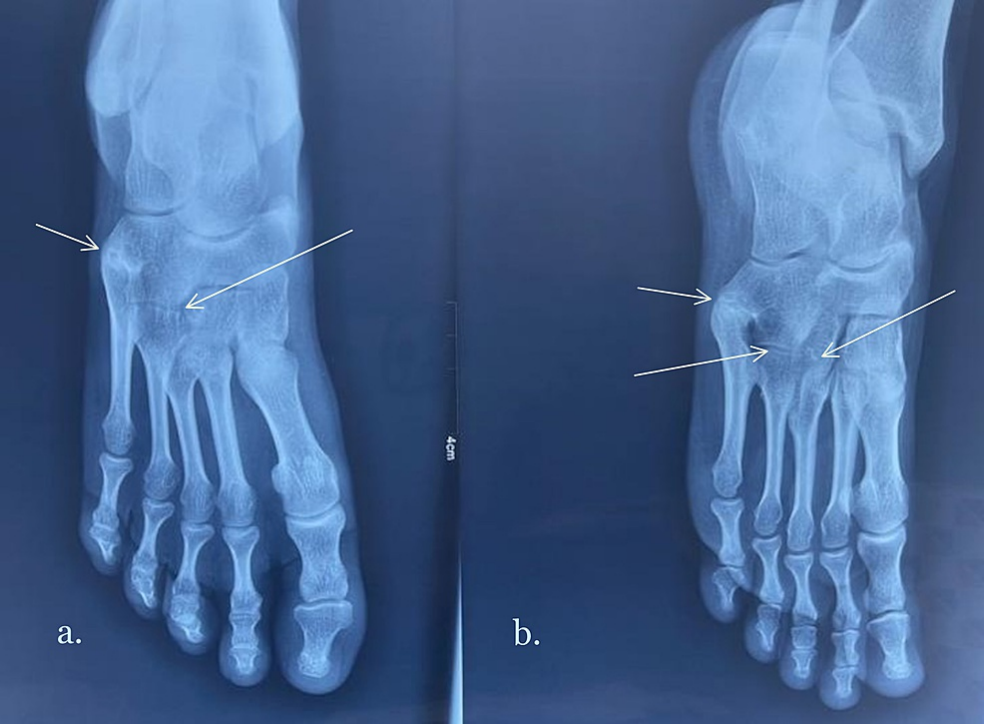
Anteroposterior (a) and oblique (b) radiographs of the right foot showing the tarsometatarsal coalitions.

Radiographs of her right foot were obtained, which revealed a coalition of the base of the fourth and fifth metatarsals with the cuboid bone, as well as a coalition of the third metatarsal with the lateral cuneiform (Figure 1). X-rays of the opposite foot did not reveal any abnormalities.

**Figure 2:**
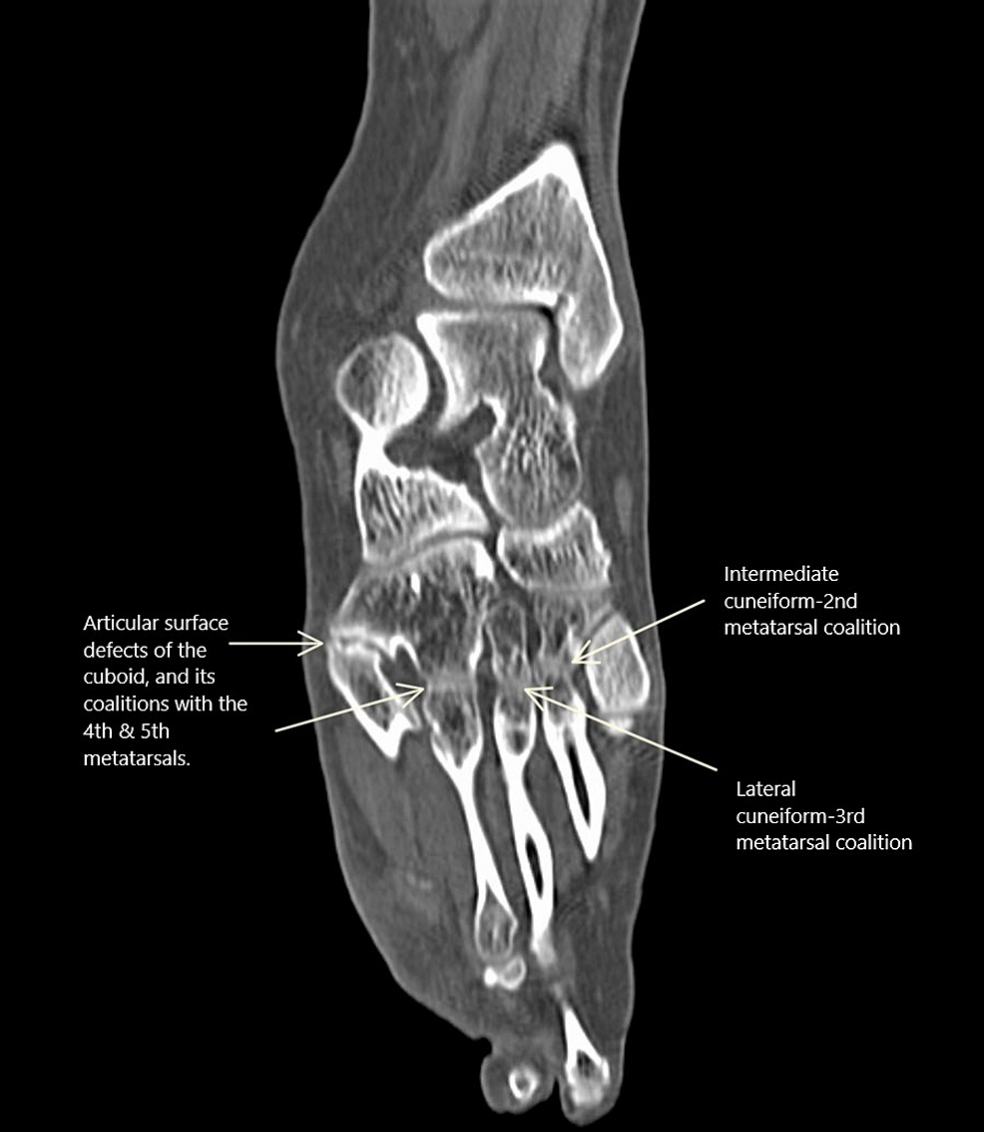
A CT scan showing the bases of the lateral four metatarsals and their respective coalitions with the cuboid, lateral, and intermediate cuneiforms.

A CT scan was subsequently performed to rule out more significant subtalar/calcaneonavicular coalition(s). The CT scan revealed the X-ray findings in greater detail; and confirmed another coalition between the second metatarsal and the intermediate cuneiform (Figure 2).

The patient was managed conservatively by a short course of non-steroidal anti-inflammatory drugs and shoe wear modification. She had been using a pair of slippers (common in the Indian subcontinent) for most of her long walks every day. Switching from slippers to low-heeled, well-fitting sports shoes considerably improved her symptoms in a few weeks. Because she showed a favorable response to treatment at her three-month follow-up, further interventions were deemed unnecessary at this stage.

## Discussion

Tarsometatarsal coalitions have seldom been described in the orthopedic literature. The handful of reports published so far have described different variations of tarsometatarsal coalitions involving one of the cuneiforms [2-4], with the opposing metatarsal respectively.

A published report by Kobayashi et al. [6] described an even rarer fifth metatarsal-cuboid coalition that needed surgical intervention after the failure of conservative treatment.

Fujishiro et al. [4] proposed that as in tarsal coalitions, patients with complete tarsometatarsal coalitions (radiological evidence of bony fusion) might be less symptomatic than those with partial coalitions (also known as non-osseous coalitions). This has also been discussed in a later case report by Stevens et al. [5], who further suggested that non-osseous coalitions should be suspected in patients diagnosed with a midfoot sprain who remain symptomatic after adequate conservative treatment. Persistently painful coalitions have been managed surgically by arthrodesis across the involved joint [5,6].

These osseous and non-osseous coalitions may be much more common than previously thought, although clinical presentation with a symptomatic foot is uncommon [5]. Indeed, descriptions of such coalitions have appeared in anthropology literature more often than in clinical literature. Regan et al. [7], while examining the remains of populations from North America and Japan, found that the incidence of third metatarsal-third cuneiform articular surface defects or coalitions was surprisingly common, with frequencies ranging from 12-20% in North American and 0-11.9% in Japanese populations.

**Figure 3:**
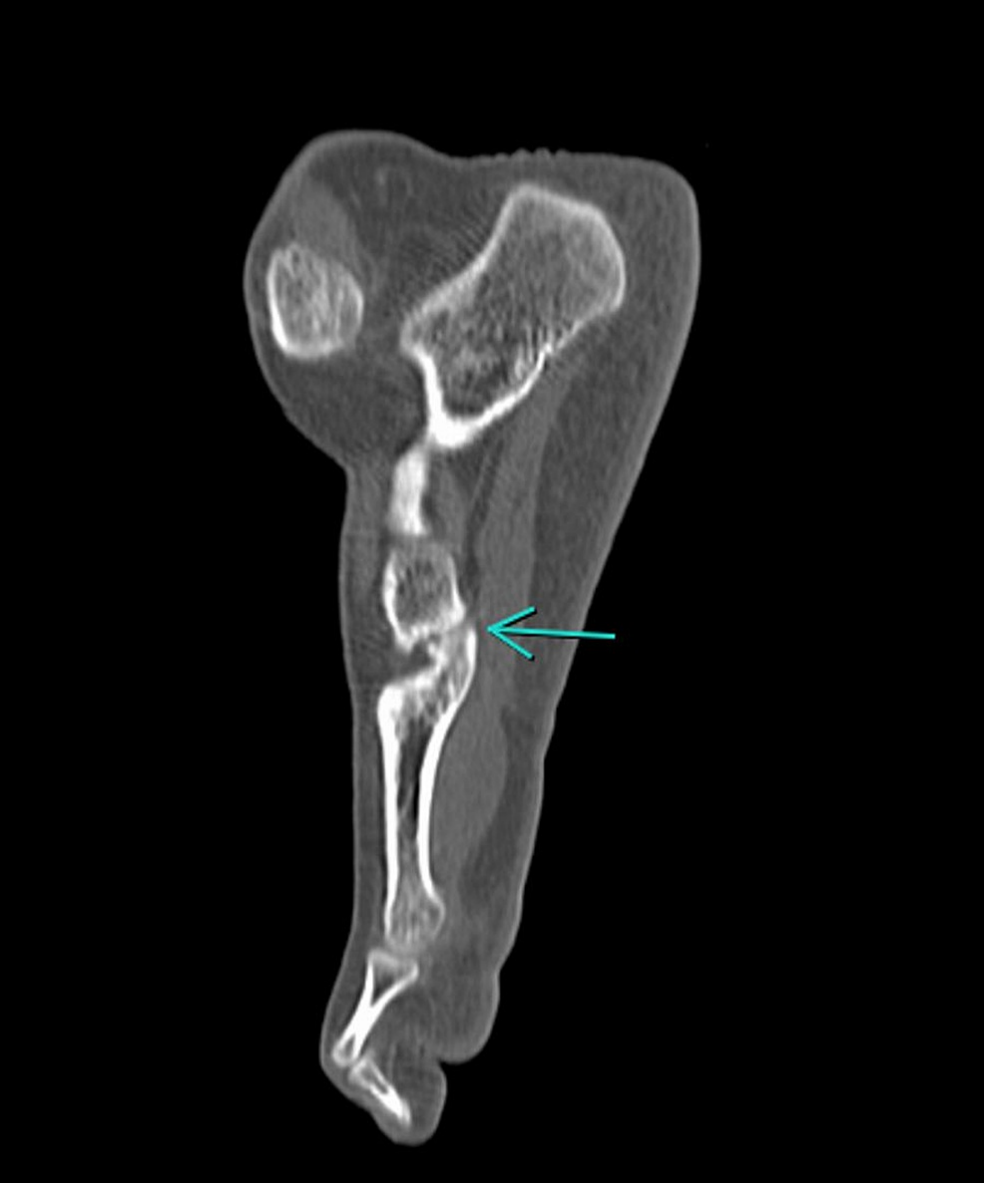
As described in the anthropology literature, the coalition appears to be more complete along the plantar aspect of the joints involved, with opposing bone surfaces curving toward each other (seen here: fifth metatarsal-cuboid coalition).

This description of articular surface defects in these tarsometatarsal coalitions bears a striking resemblance to the radiological picture of our patient. Her CT offered evidence of a similar morphology of tarsometatarsal coalition, as described by Regan et al. The cuboid and the fifth metatarsal were curved toward one another, and the tarsometatarsal coalitions were predominantly on the plantar side of the joints, just as they have been found in skeletal remains studied by anthropologists (Figure 3).

The frequent finding of such lesions in anthropology literature, as opposed to their rarity in clinical practice, suggests that most of them might be insignificant and probably do not alter the biomechanics of the foot, unlike the well-described calcaneonavicular, and talocalcaneal coalitions [8-10].

To our knowledge, ours is the only case described in the literature with coalitions across four tarsometatarsal joints (fifth metatarsal-cuboid, fourth metatarsal-cuboid, third metatarsal-lateral cuneiform, and second metatarsal-intermediate cuneiform). The fifth metatarsal-cuboid coalition was not completely ossified and was a non-osseous coalition. The other three coalitions were osseous, as confirmed by CT scan images. Despite the non-osseous nature of one of the coalitions, our patient responded to conservative treatment and was satisfied at her three-month visit. However, as our patient is young (16 years old), a long-term follow-up would be needed to ascertain if the non-osseous coalition leads to secondary osteoarthritis of the fifth metatarsal-cuboid joint. Furthermore, as the tarsal coalition has been described as a non-metric trait [7], we had hoped to screen our patient’s parents for a similar anomaly. However, we could not motivate them to undergo radiographic evaluation. Both of them were asymptomatic and did not have complaints related to their feet.

## Conclusions

Tarsometatarsal coalitions have sporadically sprung up in the orthopedic literature over the past three decades, with all authors recognizing the rarity of its incidence in clinical practice. However, based on descriptions by anthropologists in the past, tarsometatarsal coalitions might be more common than previously thought. Its rare appearance in clinical practice points to the possibility that most of these lesions might be asymptomatic and inconsequential to normal foot biomechanics.

While the possibility of an orthopedist facing a symptomatic tarsometatarsal coalition is rare in routine practice, it would be helpful to be aware of such lesions when confronted with an innocuous midfoot sprain unresponsive to conservative treatment. Persistent pain from a non-osseous coalition often resolves following arthrodesis of the involved joint.
